# Supplementary figures and images for: The mechanical influence of densification on epithelial architecture
Source: PLoS Comput Biol. 2024 Apr 1;20(4):e1012001. doi: 10.1371/journal.pcbi.1012001 (PMC11008847; doi:10.1371/journal.pcbi.1012001)

**SUPPLEMENTAL FIGURE 1 Cammarota *et al.***

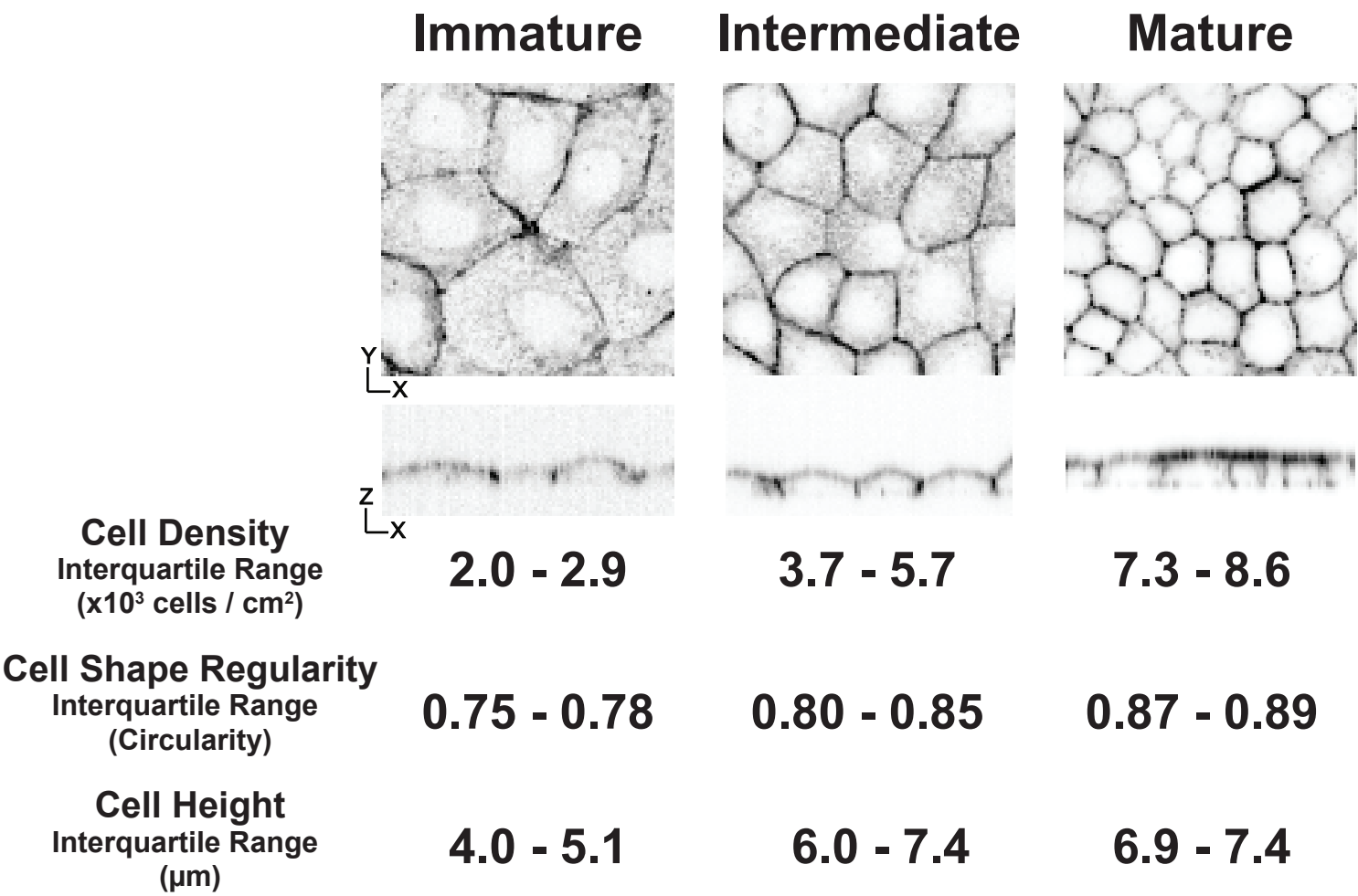

Supplement: S1 Fig — Representative confocal micrographs summarizing the distinct epithelial architecture categories made by cultured MDCK epithelial cells along with their defining average density, shape regularity in the apical plane, and height values. (PDF) [file pcbi.1012001.s001.pdf]

SUPPLEMENTAL FIGURE 2 Cammarota *et al.*

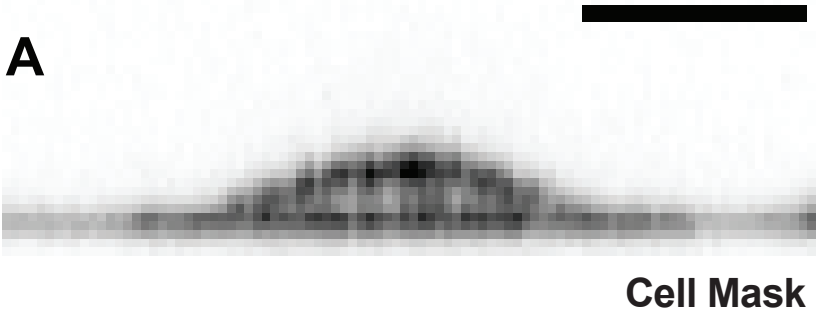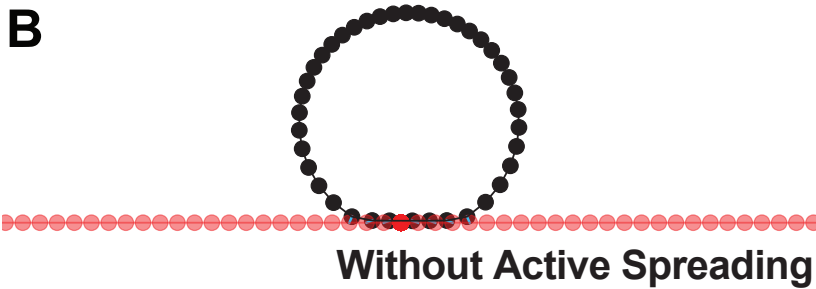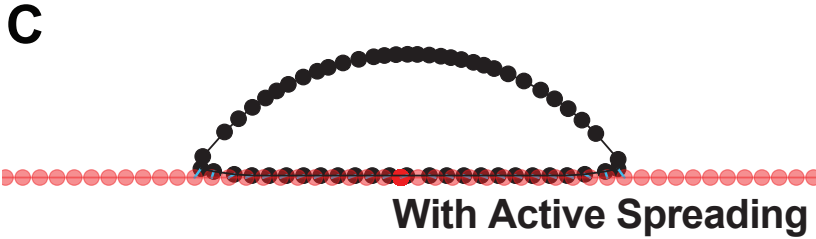

Supplement: S2 Fig — A) A representative timepoint from live confocal imaging of MDCK cells stained with CellMask upon initial culture plating shows that single cells dropped onto a substrate spread. Scale bar = 20 μm. B and C) Images of the stable shapes formed by single simulated cells without (B) and with (C) active spreading implemented. (PDF) [file pcbi.1012001.s002.pdf]

SUPPLEMENTAL FIGURE 3 Cammarota *et al.*

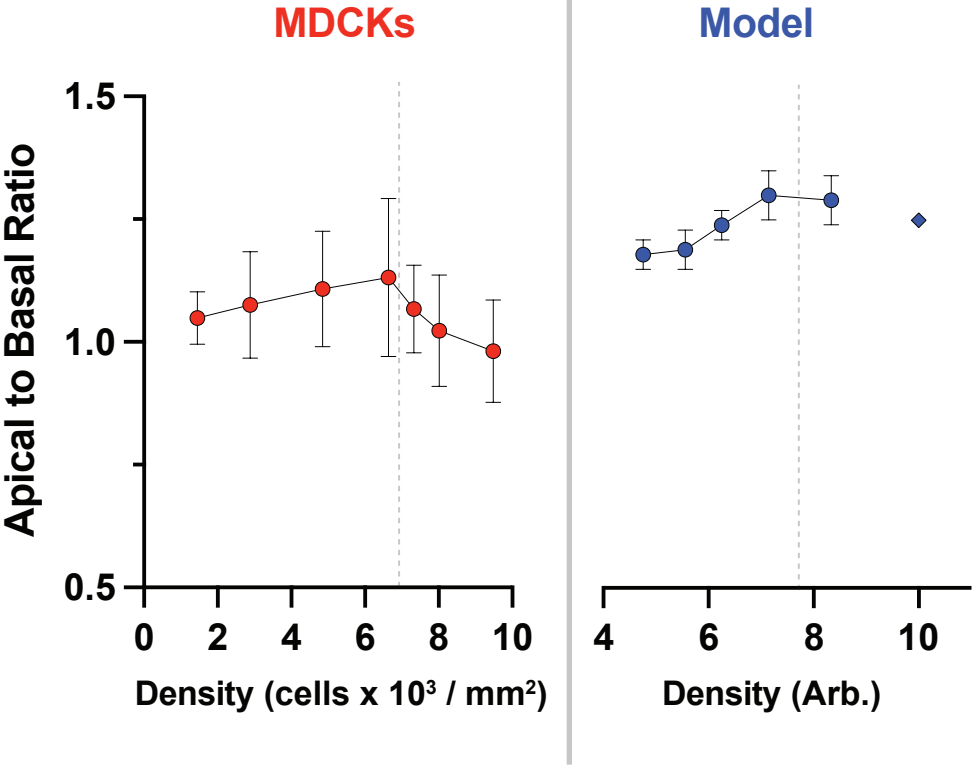

Supplement: S3 Fig — The ratio of the apical surface length to the basal surface length of simulated MDCK cells (left) grows as a function of density. Dashed line indicates the density transition between Intermediate and Mature architectures. The same ratio for modeled cells (right). Dashed line indicates predicted transition between Intermediate and Mature architectures. (PDF) [file pcbi.1012001.s003.pdf]

**SUPPLEMENTAL FIGURE 4 Cammarota *et al.***

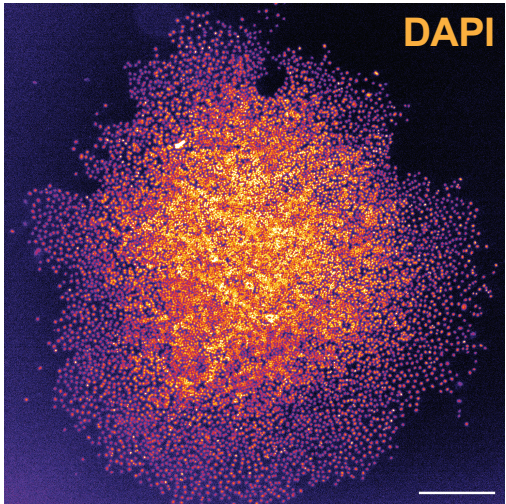

Supplement: S4 Fig — A confocal micrograph showing the distribution of nuclei (fixed DNA staining) in a colony of cultured MDCK cells. This image, also in Fig 4B, is shown here in a heatmap lookup table to emphasize the gradient of cell density starting from the colony center. (PDF) [file pcbi.1012001.s004.pdf]

# SUPPLEMENTAL FIGURE 5 Cammarota *et al.*

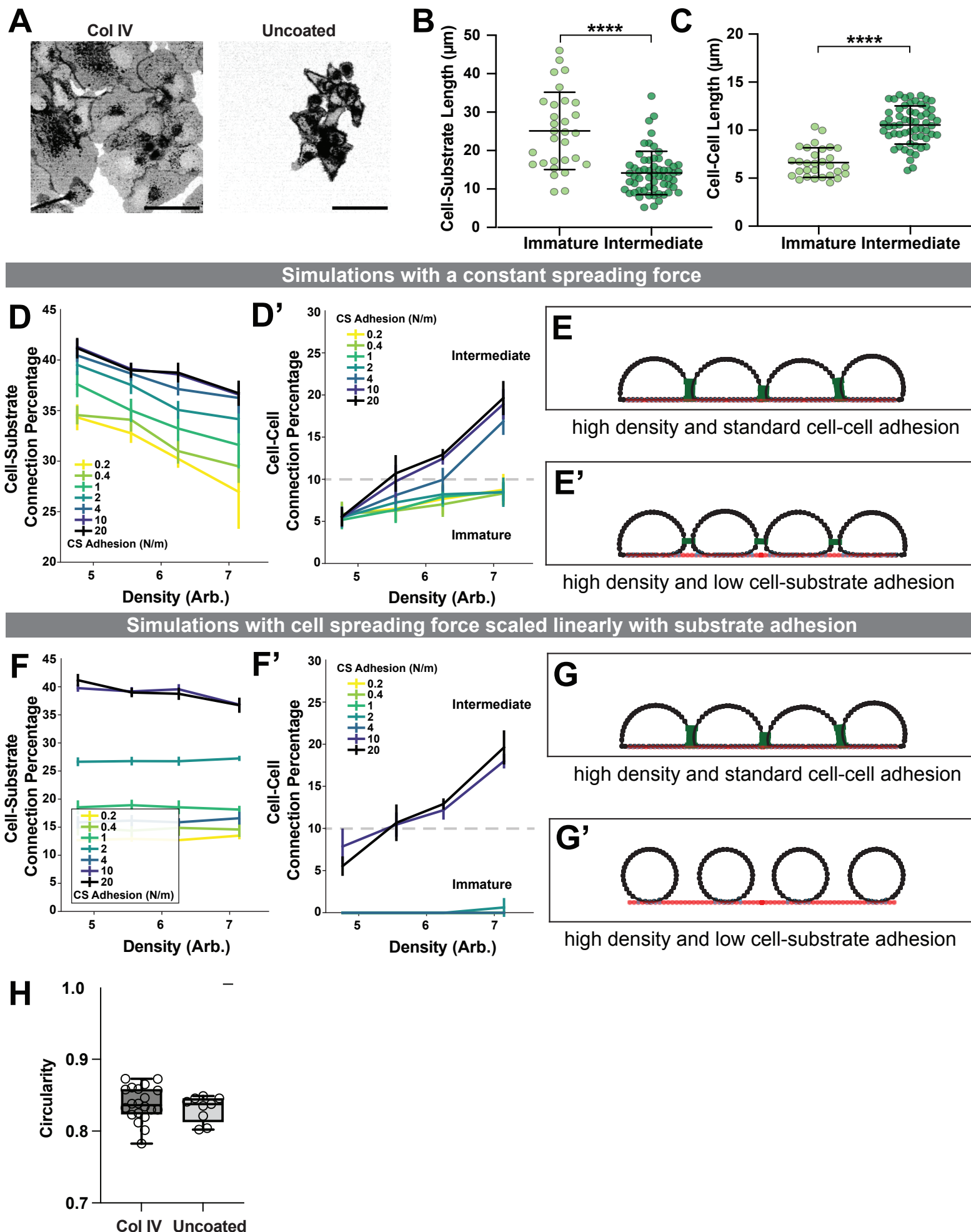

Supplement: S5 Fig — A) Cells grown on collagen spread to cover area of the substrate that is ~5 times larger cells than cells grown on an uncoated substrate. Representative confocal micrographs z-stacks of cells stained with CellMask grown on ColIagen IV and uncoated glass coverslips. Scale bar = 50 μm. B) Cell-substrate and C) cell-cell contact lengths of cultured MDCK cells decrease and increase respectively as layers transition from immature and intermediate architectures. D-G) Reducing cell-substrate adhesion strength reduces the number of cell-substrate connections in simulations with different implementations of cell spreading force. D) Cell-substrate connections decrease and D’) Cell-cell connections increase as a function of density at all substrate adhesion strengths with a constant spreading force. The dashed line (D’) represents the connection percentage required for Intermediate architectures to arise in the model. E) Final, stable equilibrium state of simulations with standard (E) and low (E’) cell-substrate adhesion. F) Cell-substrate connections decrease and F’) Cell-cell connections increase as a function of density at all substrate adhesion strengths when cell spreading force is scaled linearly with substrate adhesion. The dashed line (F’) represents the connection percentage required for Intermediate architectures to arise in the model. G) Final, stable equilibrium state of simulations with standard (G) and low (G’) cell-substrate adhesion. H) Cell shape regularity (with respect to the tissue surface) of cultured cells is not impacted by the presence of collagen on the substrate. Only Intermediate layers are shown. p = 0.4692, Significance was determined using an unpaired two-tailed Student’s t test. (PDF) [file pcbi.1012001.s005.pdf]

SUPPLEMENTAL FIGURE 6 Cammarota *et al.*

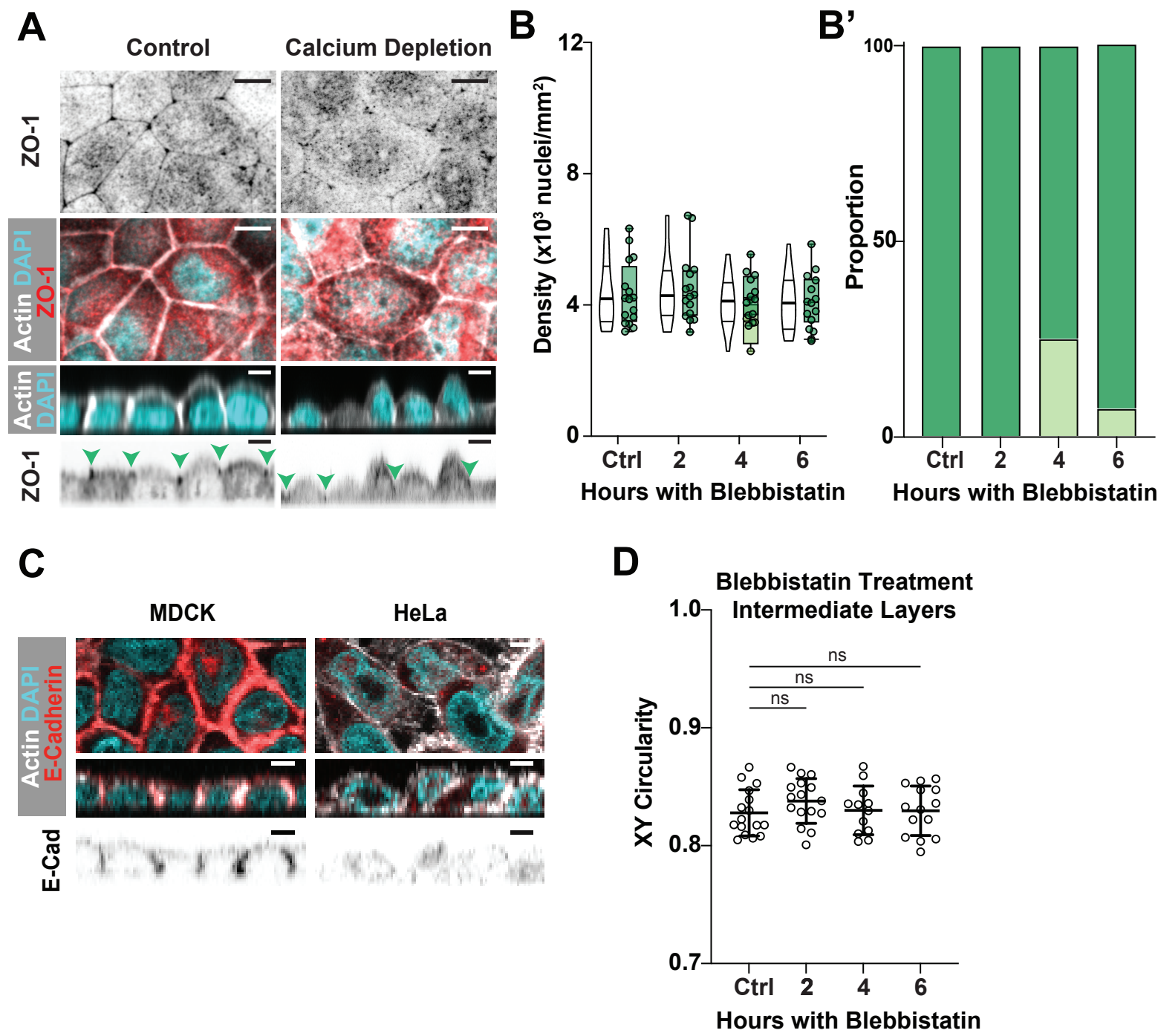

Supplement: S6 Fig — Confocal light imaging of immunostained, fixed cells shows that A) ZO-1 localizes to cell-cell junctions apically (green arrows) in mature cultured MDCK cells. Scale bars = 10 μm. After calcium depletion for 3 hrs, cells form a domed morphology. Weak ZO-1 immunoreactivity (green arrows) is evident in these cells. B) Blebbistatin does not affect the presence Intermediate architectures at the densities tested. C) MDCK cells express E-cadherin at cell-cell borders, while E-cadherin is not expressed in HeLa cells. Scale bars = 10 μm. D) Cell shape regularity (with respect to the tissue surface) is not impacted by Blebbistatin treatment. Only Intermediate architectures are shown. p values left to right: p = 0.1495, p = 0.7866, p = 0.8101. Significance was determined using an unpaired two-tailed Student’s t test. (PDF) [file pcbi.1012001.s006.pdf]

SUPPLEMENTAL FIGURE 7 Cammarota *et al.*

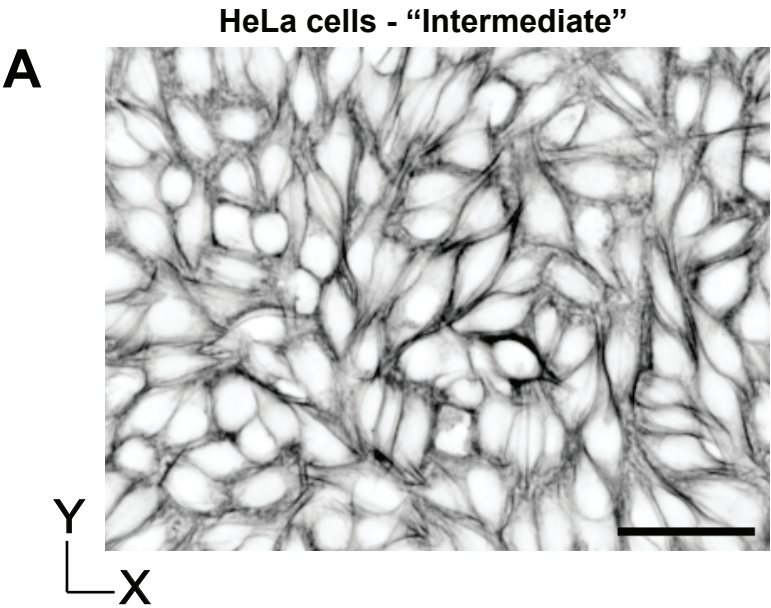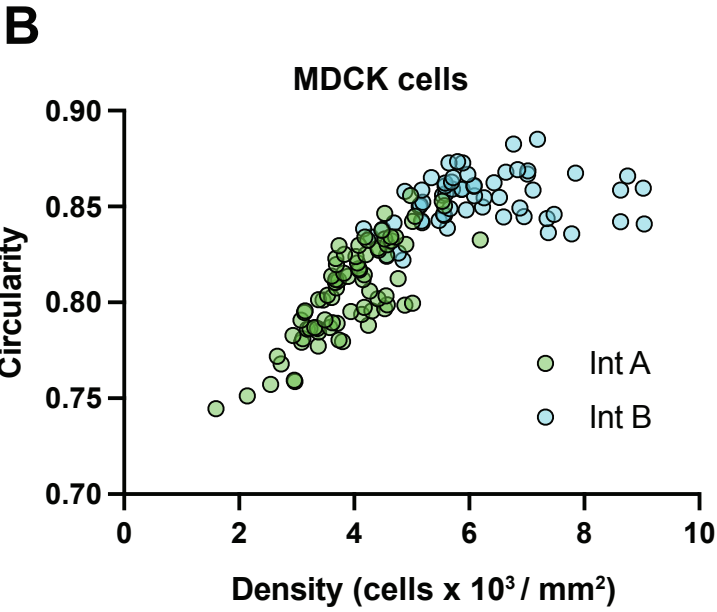

Supplement: S7 Fig — MDCK cells become regular at the Intermediate A- Intermediate B transition. A) HeLa cells exhibit spindle-like cell morphologies even in layers classed as Intermediate by our image analysis pipeline ALAn. Representative image. Scale bar = 20 μm. B) MDCK cell shape regularity (with respect to the tissue surface) is significantly lower in Intermediate A architectures in comparison to Intermediate B architectures. p < 0.0001. Significance was determined using an unpaired two-tailed Student’s t test. (PDF) [file pcbi.1012001.s007.pdf]

SUPPLEMENTAL FIGURE 8 Cammarota *et al.*

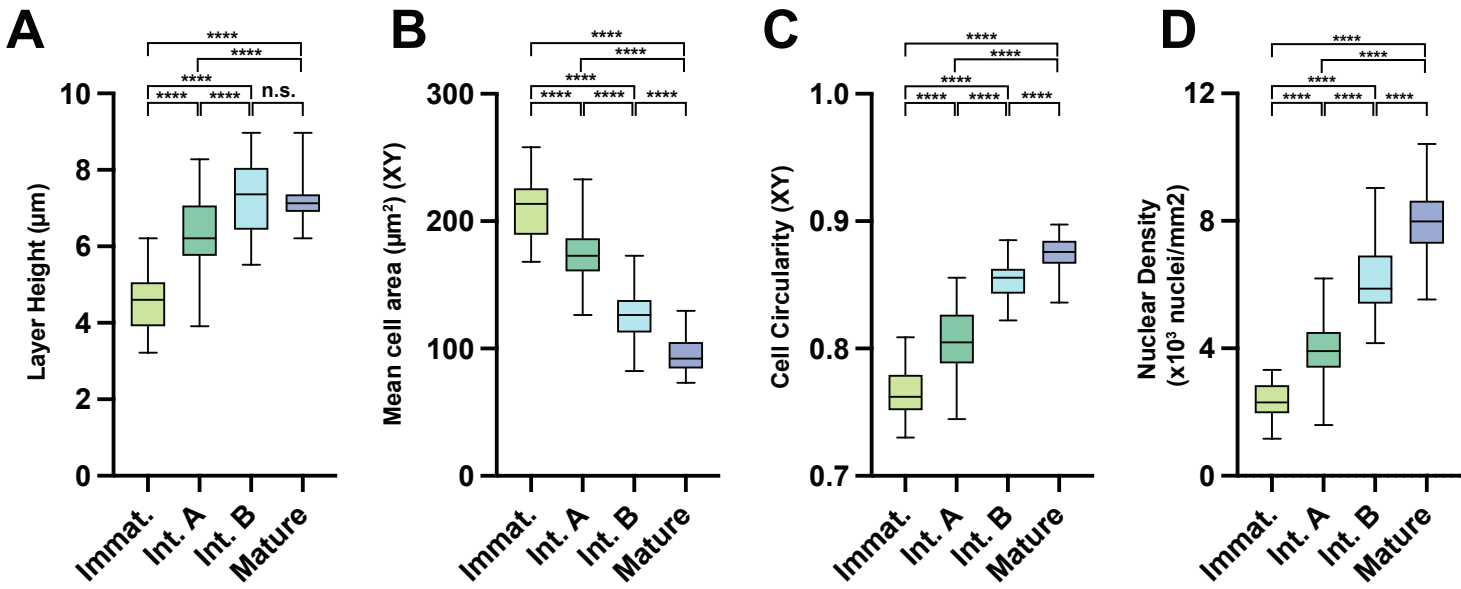

Supplement: S8 Fig — A) Intermediate B layers (7.3 μm) are taller than Intermediate A layers (6.3 μm). P values from left to right: p < 0.0001, p < 0.0001, p < 0.0001, p < 0.0001, p < 0.0001, p = 0.4497. B) Mean cell area is smaller in Intermediate B layers (126 μm2) than Intermediate A layers (173 μm2). All p values are p < 0.0001. C) Cells are more regular in intermediate B layers (0.85) than in Intermediate A layers (0.81). All p values are p < 0.0001. D) Intermediate B layers (6.16x10^3^ cells/mm2) are denser than Intermediate A layers (3.93x10^3^ cells/mm2). All p values are p < 0.0001. E) Intermediate B sublayers (6.5 μm) drawn from Intermediate A full layers are taller than Intermediate A sublayers (5.7 μm) drawn from the same population. P values from left to right: p < 0.0001, p < 0.0001, p = 0.3332. F) Intermediate B sublayers (7.1 μm) drawn from Intermediate B full layers are taller than Intermediate A sublayers (5.8 μm) drawn from the same population. p values from left to right: p = 0.4664, p < 0.0001, p < 0.0001. (PDF) [file pcbi.1012001.s008.pdf]
