# Supplementary material for: The mechanical influence of densification on epithelial architecture
Source: PLoS Comput Biol. 2024 Apr 1;20(4):e1012001. doi: 10.1371/journal.pcbi.1012001 (PMC11008847; doi:10.1371/journal.pcbi.1012001)
Supplement: Attachment — Submitted filename: PLOSComputationBiology_Mechanical Influence of densification.odt [file pcbi.1012001.s015.odt]

The manuscript aims at dissecting the role of cell density and cell-cell adhesion on the morphology of epithelial cells. This is achieved by combining a computational model with experimental results. The model is based on a finite discretization of the cell profile (in the Z plane), accounting for both the cell-cell adhesion and cell-substrate adhesion. This work closely build on top of a recent work published by the same authors where they present an automated tool to classify epithelial monolayers architectures. 
The manuscript is well written and easy to follow. I believe that the work is relevant for the biophysics and biomechanics communities and I would suggest its publication on PLOS Computational Biology. Yet, I have few concerns that I would like to authors to address upon publication, if possible. 

Major points:
1) Half of the introduction reads as a summary of the recent work published by the same authors. While the work closely build on top of the recent results, I think that the introduction would benefit from a re-writing to focus more on the scientific questions that the current manuscript is aiming at answering, what are the current gaps and how this work is going to address it.
2) Line 163: it is unclear to me why the author expected the cells to assume a flat configuration rather than a sphere.
3) Figure 2B and B’, do the cells start as individual spheres? If the cells in B’ would start from a flat configuration, would they still converge to the configuration shown in figure B’?
4) Line 239: The authors claim that the findings suggest that densification is sufficient to drive the transition from immature to Intermediate architecture. Isn’t active cell spreading also involved? If the inhibition of the feedback loop (i.e. the active cells spreading doesn’t decrease when the cell is in contact with another cell) was not present, would the monolayer assume an “immature” configuration  with all cells in a flat configuration?
5) Line 279 “Upper bound spreading condition”: what does this refer to?
6) it is not clear to me the importance of IntB. At line 467 in the discussion section the authors claim that “IntB relies on cell-cell adhesion and they propose that it represents the initial apical-basal architecture that is special to epithelial”. Yet, HeLa cells without E-cad show this phase. Why? Do the authors have some experimental results on a non-epithelial cells to show that it doesn’t possess this IntB phase?
7) What is the impact of Calcium on ZO-1?
8) The substrate has been modelled as a rigid body, what would be the impact of accounting for the elastic (or even more precisely the viscoelastic) response of it? The substrate mechanical properties is closely related to cell spreading, how does the model account for it in the PDMS simulation?

Minor points:
1) The abstract reads a lot as a continuation of the previous work. As a personal preference, I would make it more impersonal without referring too much explicitly at author’s previous work.
2) Line 38: “special” meaning unique to epithelial cell? Would it be possible to explain it in few words here.
3) I personally find the use of A, A’.. B, B’ in figures’ panels a bit confusing. I would use A, B, C… in order.
4) Line 125: “new transition”, can you add a sentence that explains what it is meant?
5) Line 143: Does “modelled as a fixed line”  mean that the substrate is a rigid body non-deformable body?
6) Line 227: reference with different style
