# Supplementary material for: The mechanical influence of densification on epithelial architecture
Source: PLoS Comput Biol. 2024 Apr 1;20(4):e1012001. doi: 10.1371/journal.pcbi.1012001 (PMC11008847; doi:10.1371/journal.pcbi.1012001)
Supplement: Attachment — Submitted filename: Response to Reviewers.docx [file pcbi.1012001.s016.docx]

We are grateful to the reviewers for their thorough and careful reviews of our manuscript. We’ve taken these reviews into account in preparing a revised version, which we believe to be substantially improved. Our responses to specific comments are described in detail below:

**Reviewer #1:**

The authors present a cell shape simulator that recapitulates several salient features cell architecture during assembly and maturation of an epithelial sheet. The authors integrate this model with experimental data collected from MDCK cell cultures over a range of densities and under specific confinement. Apical-basal cell shape changes, including contact areas with neighboring cells are generated according to parameters representing biophysical properties of the cell, including cell-cell and cell-substrate adhesion energies. Cell culture experiments are carried out to modulate several of these parameters and compare the results with analogous model perturbations. Overall, the model is interesting and has considerable potential in future applications. The physical role of confinement seems a prerequisite for the formation of a simple single-layered cell sheet modeled by MDCK and even HeLa cells with limited roles for cell-cell adhesion. This later point is surprising, but may be reflect low cell density initial conditions of their model. Future improvements on this model may be able to incorporate epithelial polarity that might enable the model to generate fully mature cell architectures. Overall, the manuscript is well written but contains several oversimplifications of the cell biology of adhesion and the role of substrate adhesion in establishment of polarity.

Some concerns:

+ There are no references to prior simulation studies on the mechanics of epithelial sheets in the transverse dimension. Classical paper by Odell is missing as are multiple Honda and Brodland papers. More recent work by the Munro lab, especially Sherrard et al (2010) should be discussed. While they do not explicitly deal with confinement, polarity and apical-basal cell shape are represented.

We thank the reviewer for flagging this oversight, which we have addressed by adding the following discussion in a new paragraph in the introduction (page 5):

*In contrast, the connection between cell shapes within the tissue plane and the observed developmental progression of epithelialization in the apical-basal axis remains poorly understood, due in part to limitations of existing computational models. While several studies have used computational modelling to explore different mechanical contributions to the deformation of epithelial sheets in this axis, these have focused primarily on gastrulation and similar processes, neglecting cell-substrate interactions. Such studies include the pioneering work of Odell et al (15) who modelled an embryo cross-section as a ring of viscoelastic cells with interconnected vertices with active contractility in response to stretching. This work, and more recent modelling efforts (16–20) have recapitulated essential features of* Drosophila *ventral furrow formation. Other applications of such modelling have included, for example, an identification of a two-step mechanism underlying early ascidian gastrulation (21). These models capture key features of epithelial cell mechanics in the transverse dimension, including active remodeling of cytoskeletal components and heterogeneous adhesion, but tend to represent both apposed cortices in each bicellular junction by a single edge, preventing neighboring cells from coupling/uncoupling to/from one another. In particular, previous models have not addressed epithelial shape development* ab initio*, nor how fundamental mechanical factors drive epithelial layer architecture diversity.*

15. Odell, G.M., G. Oster, P. Alberch, and B. Burnside. 1981. The mechanical basis of morphogenesis: I. Epithelial folding and invagination. *Developmental Biology*. 85:446–462.

16. Eiraku, M., N. Takata, H. Ishibashi, M. Kawada, E. Sakakura, S. Okuda, K. Sekiguchi, T. Adachi, and Y. Sasai. 2011. Self-organizing optic-cup morphogenesis in three-dimensional culture. *Nature*. 472:51–56.

17. Hočevar Brezavšček, A., M. Rauzi, M. Leptin, and P. Ziherl. 2012. A Model of Epithelial Invagination Driven by Collective Mechanics of Identical Cells. *Biophysical Journal*. 103:1069–1077.

18. Polyakov, O., B. He, M. Swan, J.W. Shaevitz, M. Kaschube, and E. Wieschaus. 2014. Passive Mechanical Forces Control Cell-Shape Change during Drosophila Ventral Furrow Formation. *Biophysical Journal*. 107:998–1010.

19. Rauzi, M., U. Krzic, T.E. Saunders, M. Krajnc, P. Ziherl, L. Hufnagel, and M. Leptin. 2015. Embryo-scale tissue mechanics during Drosophila gastrulation movements. *Nat Commun*. 6:8677.

20. Štorgel, N., M. Krajnc, P. Mrak, J. Štrus, and P. Ziherl. 2016. Quantitative Morphology of Epithelial Folds. *Biophysical Journal*. 110:269–277.

21. Sherrard, K., F. Robin, P. Lemaire, and E. Munro. 2010. Sequential Activation of Apical and Basolateral Contractility Drives Ascidian Endoderm Invagination. *Current Biology*. 20:1499–1510.

+ On page 4, the authors state that "Epithelia most commonly form a monolayered architecture"... Greater care should be taken to properly place the current model into the class of "simple epithelia". There is considerable diversity in epithelial architectures (see Bragulla and Homberg, 2009).

Fair point. We’ve changed the text as follows (line 53, page 4):

*The most common epithelial architecture is a “simple” monolayer (a sheet of cells that is one cell thick), with component cells sharing a common apical-basal polarity axis, adhering closely to their lateral neighbors, and a uniform distribution of nuclei with respect to the apical-basal axis (1, 2).*

1. Bragulla, H.H., and D.G. Homberger. 2009. Structure and functions of keratin proteins in simple, stratified, keratinized and cornified epithelia. *J Anat*. 214:516–559.

2. Buckley, C.E., and D. St Johnston. 2022. Apical-basal polarity and the control of epithelial form and function. *Nat Rev Mol Cell Biol*. 23:559–577.

+ I enjoyed the "model building" introduction starting with single cells on an adhesive substrate. However, the immediate inclusion of a phenomenological feedback circuit between cell-cell and cell-substrate adhesion was premature. I would rather this have been introduced to "fix" some deficit. Can this feedback be removed?

We thank the reviewer for highlighting the lack of clarity regarding our motivation for including this in our model. We have addressed this in two ways:

First, justification of the feedback mechanism has been strengthened by the inclusion of an additional supporting reference (page 7):

30. Choi, D., Z. Gonzalez, S.Y. Ho, A. Bermudez, and N.Y.C. Lin. 2022. Cell-cell adhesion impacts epithelia response to substrate stiffness: Morphology and gene expression. *Biophysical Journal*. 121:336–346.

Secondly, the answer to the reviewer’s question is that the feedback can be removed without impacting most of the simulations described in our paper. It is most relevant at low substrate adhesion and we have added text to describe this (line 278, page 11):

*This is because the shape of a simulated cell in our model is determined largely by a balance between two parameters, spreading and the internal regulation of cell shape, which is buffered by the positive feedback between cell-cell and cell-substrate adhesion described above. Simulated cells become more circular (Figure 4B, Figures S5E,G) when this balance is shifted by weakened cell-substrate adhesion.*

+ Several additional phenomenological rules are added to the model, including contact-inhibition-of-locomotion (CIL). Formally, there is no real locomotion. Can this rule be removed? and how does it change the outcome? Do MDCK cells demonstrate CIL in culture? Can this version of CIL emerge just from competition for adhesive substrates?

**FIGURE I: Representative simulation in which contact of inhibition of spreading is not implemented.**

The reviewer raises a very good point and has led us to rethink this rule. Rather than describe this as “contact inhibition of locomotion,” we have changed the term to “contact inhibition of spreading.” Without this rule, simulate cell borders (defined by the number and position of nodes) do not remain distinct but instead “bleed” into/over one another, as shown here (Figure I).

We have rewritten the text accordingly (line 175,page 8):

*We also incorporated contact inhibition of spreading, a phenomenon similar to contact inhibition of locomotion, into our model (34). To achieve this, the spreading force drops to zero when a simulated cell is in contact with a neighbor. Contact inhibition of spreading is implemented at each edge of the cell independently, meaning that spreading continues on the other side of the cell until/unless it also contacts a neighbor.*

+ The terms for the various architectures, disordered, immature, intermediate, and mature, imply a progression akin to a mesenchymal to epithelial transition during morphogenesis. However, MDCK cells are an immortalized epithelial cell line and have little to no developmental change. Freshly plated cells already express all the proteins to generate a polarized mature epithelium, there is no progression. Model cells do not have the ability to polarize in this manner and are more representative of non-epithelial cells, such as the HeLa cells used briefly in Fig 5.

We absolutely agree that the model cells do not polarize and are more like HeLa cells in that regard.

While we see where the reviewer is coming from, we respectfully disagree with the idea that MDCK cells do not undergo relevant biological changes – akin to development - as they densify. We do not claim to see MET in our system, but we do think that our observations and those of others show that some aspects of epithelial architecture (and thus function) are under mechanical regulation.

1) MDCK cells do not develop tight junctions below a certain density (Dawney & Cammarota *et al.* Mol. Biol. Cell 2023), and we consider these junctions to be characteristic of a polarized, mature epithelium.
2) Consistent with this is a report showing that the tight junction component claudin-2 is expressed in confluent but not subconfluent cells (Amoozadeh *et al* Am J Physiol Cell Physiol. 2018). This work indicates that MDCK cells may not have all of the genes necessary to form a mature polarized epithelium when freshly plated.
3) N-glycosylation of E-Cadherin and other proteins is regulated by GPT (dolichol-P-dependent N-acetylglucosamine-1-phosphate-transferase), which is reported to have lower expression and activity in dense MDCK cultures than in sparse cultures (Sengupta *et al.* J Cell Sci. 2013). N-glycosylation is thought to reduce E-cadherin-mediated adhesion, highlighting the fact expression is only one indicator of functional output; included among other parameters are protein localization and modification.
4) The apical brush border, which might be considered another marker for a mature epithelium, becomes significantly more pronounced as MDCK cells densify (Dawney & Cammarota *et al.* Mol. Biol. Cell 2023).
5) Several groups have also reported changes in MDCK cell gene expression - at both the protein and mRNA levels - under different mechanical conditions. Unsurprisingly, these studies have demonstrated different expression profiles in adherent versus suspension cultures, though these changes also represent adaptation over time (Ye *et al.* Mol. Omics 2021 and Kluge *et al.* Vaccine 2015). More relevant to our work is another study showing extensive changes in gene expression between MDCK cells at the time of seeding and after 11 days of 2D culture (Wang *et al.* iScience 2020). Among the upregulated gene sets are those involved in cell adhesion and polarity.
6) Our group has performed preliminary RNASeq analysis on the different layer types. We also find changes in gene expression as architectures transition, but these results are not yet validated.

+ Could the authors clarify the connection between confinement and density? It seems that density is the product of confinement, and growth, division, or motility. Can density be achieved without this?

If we understand the question correctly (I hope that we do!), the reviewer is asking if there is a pathway to densification that does not rely on confinement. We haven’t been able to come up with such a mechanism. However, the second reviewer raised a point we think is relevant to this question, which we have sought to address through an additional simulation study (see Reviewer 2 comment 3, Figure II below). We also added a clarification to the text that confinement could come from sources other than proliferation (line 221, page 10):

*The most obvious answer is cell division (other routes of confinement and therefore densification could be the application of extrinsic compressive forces, or collision with other cell colonies).*

+ On page 10, the authors state that the margin of an epithelial patch is "pushed out" by proliferative pressure. Wouldn't there be a "free-edge" response at the margin that would initiate outward directed cell traction? Such traction would necessarily add to the tension across the dorsal surface of the cell sheet and "smooth" the surface.

Yes, we agree with this point. We’ve adjusted the text as follows (line 232, page 10):

*…the colony edge expands outward through a combination of proliferation throughout the colony and active spreading by outer cells.*

+ The authors use uncoated wells as a way to reduce substrate adhesion. However, MDCK cells are well known to synthesize their own ECM over time (Yu et al, MBoC 2004). Furthermore, the 10% FBS culture media contains sufficient fibronectin and vitronectin for the cells to assemble more ECM (Hayman et al, 1985). Would it be possible to increase substrate adhesion by providing cells with additional soluble ECM?

This is a great idea and one we are enthusiastic about pursuing. We absolutely intend to explore the role of substrate adhesion in apical-basal architecture more thoroughly in the future. Further experiments we’d like to try along this line include genetic manipulation of ECM receptors. As discussed in the paper, our efforts to date have been frustrated by our inability to measure substrate attachment directly. Without a solution to this problem we cannot be sure whether providing soluble ECM components will promote substrate adhesion (we imagine this to be the case, but are also mindful that tight junctions appear in our Intermediate layers, presenting a potential barrier) and if so, to what extent. An additional complication is the possibility that these factors might trigger signaling events independent of adhesion. For these reasons, we want to reserve this experiment for a more complete future study.

+ Calcium withdrawal exposes hyper-adhesivity in MDCK cells. This requires the formation of desmosomes. See Conway group or Garrod group's work.

We made sure to perform our calcium withdrawal experiments within the timescale that interpretation would not be confounded by desmosome formation. We’ve clarified this point in the text as follows (line 357, page 14):

*Although prolonged calcium withdrawal (~6 days) is associated with desmosome-dependent hyper-adhesivity (52) our measurements were performed after only 3 hours and therefore avoided that concern.*

Minor issues:

+ As a note: it would be helpful if the authors could put their results in the context of recent paper from the Gardel group (Devany et al, 2023). The difference in cell shapes may be related to changes in the cell cycle.

We agree that this was oversight on our part. We have added a paragraph to the discussion (line 496, page 19):

*Recent work revealed that MDCK cells follow a ‘sizer’ behavior; smaller cells will grow more than larger cells during the cell cycle (56). Sizer behavior would cause cell sizes to tend toward homogeneity, and in turn cell shape regularity. In agreement with this prediction, our work shows that cell shapes (with respect to the tissue plane) become more regular as MDCK cells densify.*

56. Devany, J., M.J. Falk, L.J. Holt, A. Murugan, and M.L. Gardel. 2023. Epithelial tissue confinement inhibits cell growth and leads to volume-reducing divisions. Developmental Cell. 58:1462-1476.e8.

+ On page 14, Yeh-Shiu et al, 2004 is referenced without an inline cite.

Thank you for catching this error. We’ve fixed the citation.

**Reviewer #2:**

The manuscript aims at dissecting the role of cell density and cell-cell adhesion on the morphology of epithelial cells. This is achieved by combining a computational model with experimental results. The model is based on a finite discretization of the cell profile (in the Z plane), accounting for both the cell-cell adhesion and cell-substrate adhesion. This work closely build on top of a recent work published by the same authors where they present an automated tool to classify epithelial monolayers architectures.

The manuscript is well written and easy to follow. I believe that the work is relevant for the biophysics and biomechanics communities and I would suggest its publication on PLOS Computational Biology. Yet, I have few concerns that I would like to authors to address upon publication, if possible.

Major points:

1) Half of the introduction reads as a summary of the recent work published by the same authors. While the work closely build on top of the recent results, I think that the introduction would benefit from a re-writing to focus more on the scientific questions that the current manuscript is aiming at answering, what are the current gaps and how this work is going to address it.

We have substantially rewritten the introduction of the paper in response to both reviewer’s suggestions (lines 52-127, pages 4-6).

2) Line 163: it is unclear to me why the author expected the cells to assume a flat configuration rather than a sphere.

We built our model first to recapitulate the behavior of MDCK cells in simple configurations so that we could make predictions about more complex configurations. For that reason, we used cultured cells to govern our expectations for the model. We have slightly rewritten the manuscript to describe the plating condition more fully and direct readers to a reconstructed confocal micrograph of an MDCK cell in culture in Figure S2A (line 164, page 8):

*Our initial expectation was that cell-substrate adhesion would be sufficient to promote the flat shape of isolated cells cultured on a substrate (Figure S2A)…*

3) Figure 2B and B’, do the cells start as individual spheres? If the cells in B’ would start from a flat configuration, would they still converge to the configuration shown in figure B’?

This is a cool, thought-provoking idea for a simulated experiment, and we think it is relevant to a point about confinement and densification made by Reviewer #1. We struggled to devise a way to carry it out but ultimately came up with the following: We started the simulation with four flat simulated cells on a substrate long enough to support them in that configuration, *then* reduced the length of the substrate. Frames from the resulting simulation are shown here (Figure II).

In this simulation, the interior cells do *not* become taller (as in Figure B’) whereas the two exterior cells do. Unfortunately, we are not sure how to interpret this result. It does not seem to us to be physiologically relevant, as the outside cells find themselves protruding into aether. We speculate that simulated tissue would reach the B’ configuration if the component cells were more viscous. This could potentially be achieved by active remodeling of adhesions (meaning turnover of cell-cell and cell-substrate junctions as we recently studied in Nestor-Bergmann A.,… Fletcher A.G.,… *et al.* PloS Comp Biol 2022) or through cell divisions, which can provide active stress in an epithelial monolayer as in Devany *et al.* PNAS 2021).

**FIGURE II: Simulation of an immature layer subjected to sudden confinement.**

4) Line 239: The authors claim that the findings suggest that densification is sufficient to drive the transition from immature to Intermediate architecture. Isn’t active cell spreading also involved? If the inhibition of the feedback loop (i.e. the active cells spreading doesn’t decrease when the cell is in contact with another cell) was not present, would the monolayer assume an “immature” configuration with all cells in a flat configuration?

Yes, active spreading is also involved, and we have amended the sentence to clarify that point (line 234, page 10)

*Both theory and our simulations predict that in combination with active spreading, division pressure promotes the transition from Immature to Intermediate architecture (37).*

Unfortunately, we cannot run the suggested simulation for a technical reason described above, namely that the feedback loop is required to ensure that cells don’t “bleed through” one another (Figure I).

5) Line 279 “Upper bound spreading condition”: what does this refer to?

“Upper bound spreading condition” is referred to earlier in the manuscript as the first model of active spreading; the spreading force is held constant as a function of substrate adhesion. We have copied the two relevant sections and highlighted the use of “upper bound spreading condition” here.

Original Text:

*We therefore undertook simulations using three models for active spreading: 1) the spreading force is held constant at all substrate adhesions; 2) the spreading force scales linearly with substrate adhesion; and 3) the spreading force scales non-linearly with substrate adhesion.* We consider the first condition to represent an upper bound *and the second to represent the lower bound, whereas the third most closely represents our observations in culture.*

*Spreading strength modulates the role of substrate adhesion in the transition from Immature to Intermediate architecture in our simulations. In both the nonlinear and* upper bound spreading conditions*, Intermediate architectures develop even when cell-substrate adhesion is reduced by as much as 80% (from 20 N/m to 4 N/m) (Figure 4A’, Figure S5D’). In the lower bound condition, in which spreading and adhesion have a linear relationship, Intermediate architectures are observed at 50% adhesion strength (Figure S5F’).*

We have adjusted the text as follows (line 285, page 12):

*Spreading strength modulates the role of substrate adhesion in the transition from Immature to Intermediate architecture in our simulations. In both the nonlinearly scaled and constant strength spreading conditions, Intermediate architectures develop even when cell-substrate adhesion is reduced by as much as 80% (from 20 N/m to 4 N/m) (Figure 4A’, Figure S5D’). In the linearly scaled spreading condition, Intermediate architectures are observed at 50% adhesion strength (Figure S5F’).*

6) it is not clear to me the importance of IntB. At line 467 in the discussion section the authors claim that “IntB relies on cell-cell adhesion and they propose that it represents the initial apical-basal architecture that is special to epithelial”. Yet, HeLa cells without E-cad show this phase. Why? Do the authors have some experimental results on a non-epithelial cells to show that it doesn’t possess this IntB phase?

We suspect that this comment reflects a communication failure on our part. None of the HeLa cell layers that we examined reached Intermediate B architectures even at their highest density. In working to address the reviewer’s concern we realized that the layout and legend for the original Supplemental Figure 7 are confusing and might easily lead to misinterpretation. This figure has two panels, A and B. Panel A shows the XY morphology of HeLa cells classed by our analysis tool as Intermediate. Panel B is a graph showing the relationship between cell circularity (Y axis), cell density (X axis), and layer architecture (IntA in green, IntB in blue) in MDCK cells. The fact that Panel B represents a different cell type to that used in Panel A was not made clear outside of the main text. Compounding the problem was that the two panels were positioned side-by-side. We have adjusted both the figure and legend and hope that these improvements are helpful.

7) What is the impact of Calcium on ZO-1?

We performed immunostaining of ZO-1 in Mature layers following 3 hrs of calcium depletion. We have added this data to the manuscript in Figure S5A and added the following text (line 363, page 14):

*Calcium depletion appears to diminish but not eliminate tight junctions (as marked by ZO-1 immunostaining) in these cells (Figure S6A).*

8) The substrate has been modelled as a rigid body, what would be the impact of accounting for the elastic (or even more precisely the viscoelastic) response of it? The substrate mechanical properties is closely related to cell spreading, how does the model account for it in the PDMS simulation?

We did not simulate the PDMS substrate and we regret giving this impression. Our model does not permit substrate deformation. However, as a proxy for this we did simulate different levels of cell spreading and found that spreading strength modulates the substrate adhesion at which cells can form intermediate architectures (SFig5D-G)

We and others have previously explored the effects of basement membrane viscoelasticity on epithelial dynamics (Dunn SJ, Fletcher AG, *et al* J Theor Biol. 2012) and we agree that extending our present model to the case of a viscoelastic substrate is an interesting avenue for future work.

Minor points:

1) The abstract reads a lot as a continuation of the previous work. As a personal preference, I would make it more impersonal without referring too much explicitly at author’s previous work.

We appreciate the reviewer’s feedback and have rewritten the abstract as follows:

***“****Epithelial tissues are the most abundant tissue type in animals, lining body cavities and generating compartment barriers. The function of a monolayered epithelial tissue – whether protective, secretory, absorptive, or filtrative – relies on the side-by-side arrangement of its component cells. The mechanical parameters that determine the shape of epithelial cells in the apical-basal plane are not well-understood. Epithelial tissue architecture in culture is intimately connected to cell density, and cultured layers transition between architectures as they proliferate. This prompted us to ask to what extent epithelial architecture emerges from two mechanical considerations: A) the constraints of densification and B) cell-cell adhesion, a hallmark feature of epithelial cells. To address these questions, we developed a novel polyline cell-based computational model and used it to make theoretical predictions about epithelial architecture upon changes to density and cell-cell adhesion. We tested these predictions using cultured cell experiments. Our results show that the appearance of extended lateral cell-cell borders in culture arises as a consequence of crowding – independent of cell-cell adhesion. However, cadherin-mediated cell-cell adhesion is associated with a novel architectural transition. Our results suggest that this transition represents the initial appearance of a distinctive epithelial architecture. Together our work reveals the distinct mechanical roles of densification and adhesion to epithelial layer formation and provides a novel theoretical framework to understand the less well-studied apical-basal plane of epithelial tissues.”*

2) Line 38: “special” meaning unique to epithelial cell? Would it be possible to explain it in few words here.

We chose “special” carefully because we can’t be certain that it is unique to epithelial cells. We’ve replaced the original clause with “*distinctive epithelial architecture*” and hope this edit makes our meaning clearer.

3) I personally find the use of A, A’.. B, B’ in figures’ panels a bit confusing. I would use A, B, C… in order.

While we appreciate the reviewer’s point, we’ve elected to stick with the current labels. We prefer this system because it groups experimental results together and therefore highlights meaningful comparisons.

4) Line 125: “new transition”, can you add a sentence that explains what it is meant?

We have adjusted the text as follows (line 125, page 6):

*However, we also identify two distinct morphological categories within the Intermediate architecture. Our findings indicate a new transition in confluent cell architecture within the Intermediate category that is restricted to epithelial cells.*

5) Line 143: Does “modelled as a fixed line” mean that the substrate is a rigid body non-deformable body?

The reviewer is correct. We have clarified this in the text (line 143, page 7):

*The XZ cross* section (i.e. apical-basal axis) *of each cell’s cortex is modelled by a polygon comprising many vertices or ‘nodes’; similarly, the substrate is modelled by a fixed line (rigid non-deformable body) discretized into a large number of nodes (Figure 1).*

6) Line 227: reference with different style

Thanks for catching this. Citations have now been corrected.
